# Measuring concordance and discordance between selected individual characteristics and corresponding neighborhood-level social determinants of health

**DOI:** 10.1017/cts.2024.517

**Published:** 2024-04-15

**Authors:** Ka Ming Ngai, Andrew Maroko, Saadiyah Bilal, Marcee Wilder, Lauren Gordon, Lynne D. Richardson

**Affiliations:** 1 Institute for Health Equity Research, Icahn School of Medicine at Mount Sinai, New York, NY, USA; 2 Department of Emergency Medicine, Icahn School of Medicine at Mount Sinai, New York, NY, USA; 3 Department of Population Health Science and Policy, Icahn School of Medicine at Mount Sinai, New York, NY, USA

**Keywords:** Health disparities, individual risk factors, neighborhood risk factors, social determinants of health (SDoH)

## Abstract

**Background:**

Individuals reside within communities influenced by various social determinants impacting health, which may harmonize or conflict at individual and neighborhood levels. While some experience concordant circumstances, discordance is prevalent, yet poorly understood due to the lack of a universally accepted method for quantifying it. This paper proposes a methodology to address this gap.

**Methods:**

We propose a systematic approach to operationalize concordance and discordance between individual and neighborhood social determinants, using household income (HHI) (continuous) and race/ethnicity (categorical) as examples for individual social determinants. We demonstrated our method with a small dataset that combines self-reported individual data with geocoded neighborhood level. We anticipate that the risk profiles created by either self-reported individual data or neighborhood-level data alone will differ from patterns demonstrated by typologies based on concordance and discordance.

**Results:**

In our cohort, it was revealed that 20% of patients experienced discordance between their HHIs and neighborhood characteristics. Additionally, 38% reside in racially/ethnically concordant neighborhoods, 23% in discordant ones, and 39% in neutral ones.

**Conclusion:**

Our study introduces an innovative approach to defining and quantifying the notions of concordance and discordance in individual attributes concerning neighborhood-level social determinants. It equips researchers with a valuable tool to conduct more comprehensive investigations into the intricate interplay between individuals and their environments. Ultimately, this methodology facilitates a more accurate modeling of the true impacts of social determinants on health, contributing to a deeper understanding of this complex relationship.

## Introduction

The importance of understanding social determinants of health (SDoH) to improve health outcomes and mitigate inequities is widely recognized [1]. SDoH encompass the conditions in which people are born, live, learn, work, play, worship, and age, affecting a wide range of health, functional, and quality-of-life outcomes and risks [2]. SDoH operate at both the individual and the neighborhood levels; personal factors (e.g., race, income, education) interact with neighborhood factors (e.g., social and physical environmental amenities and disamenities) [2] to impact health [3–5].

While a substantial body of literature exists on the association between self-reported individual-level SDoH and health outcomes, there is a growing interest in understanding the impact of neighborhood SDoH on health [6]. Typically, studies either utilize individual-level data to draw inferences about others with similar race/ethnicity, education, or income, or they employ neighborhood-level data to predict health risks for residents of a given area. However, people have both individual- and neighborhood-level social determinants that may confer health risks or health benefits. While these are often aligned, this is not always the case. An affluent individual may live in a neighborhood with a high median income (concordance), or an affluent individual may live in a neighborhood with a low median income (discordance). In the latter scenario, the person may experience both the health benefits of their individual income and the health risks associated with residing in an economically disadvantaged area. We hypothesize that individuals with concordant individual- and neighborhood-level characteristics may exhibit different health outcomes compared to those with discordant characteristics. Moreover, there may be interactions between individual- and neighborhood-level factors that yield health effects not readily apparent when examining these factors in isolation. Therefore, operationalized definitions of concordance and discordance are essential for exploring these effects.

In this paper, we propose a method to operationalize the concepts of concordance and discordance between individual-level and neighborhood-level social determinants, providing selected examples using household income (HHI) and race/ethnicity. We first consider HHI, a factor measured with a continuous variable, followed by race/ethnicity, a categorical variable. To illustrate our proposed method, we applied our operational definitions to a small dataset comprised of self-reported individual data paired with geocoded publicly available neighborhood-level data based on residential addresses. We anticipate that the risk profiles generated by either individual-level or neighborhood-level data alone will differ from those derived from typologies based on concordance and discordance.

## Materials and methods

### Data sources

PRIORity (*P*redicting *R*isk and *I*nvestigating *O*utcomes using Patient-*R*eported and Commun*ity*-level Social Determinants Data in Vulnerable Populations) is a prospective observational cohort study of randomly selected, high-risk (at least one chronic medical condition) adult emergency department (ED) patients who reside in New York City (NYC). The study recruited at the four highest volume EDs in our health system.

### Priority survey enrollment and data collection

A 36-item survey, using validated SDOH questions, was administered as an interview by research staff to collect individual demographic, social, financial, and environmental characteristics, including participants’ addresses. The survey is available as Appendix—Priority Enrollment Survey. Patients were eligible to participate if they were as follows: (i) adult ≥ 18 years old, (ii) in the ED during enrollment hours, (iii) had at least one of these common chronic conditions—hypertension, heart failure, asthma/chronic obstructive pulmonary disease, diabetes, or kidney failure—and (iv) able and willing to consent in Chinese, Spanish, or English. The survey was readministered to all participants by phone at 6 and 12 months. This study was approved by our institution’s review board, and all participants provided written informed consent prior to enrollment in the study.

For purposes of our example, we use one continuous variable, self-reported HHI, and one non-ordinal categorical variable, self-reported race/ethnicity, from the baseline survey administered during the initial ED visit. Participant survey data include race/ethnicity categories (respondents may choose multiple categories) and HHI presented in $20,000 intervals. We chose these two variables for our example because (i) there is publicly available, comparable neighborhood-level data, (ii) there is strong evidence that they are each associated with multiple health outcomes at both the individual and the neighborhood levels, and (iii) they are the most familiar components of neighborhood disadvantage.

### Definition of neighborhood

We defined a neighborhood as a walkable distance along pedestrian-accessible networks within a ¼ mile radius around each participant’s home address. This definition offers a more realistic and nuanced portrayal of “neighborhood” in urban environments, which is more reflective of a participant’s lived experience than more commonly used definitions such as a simple Euclidean buffer (e.g., a circle with a ¼ mile radius) or simple containment (e.g., the census tract or ZIP code in which the home address is located) [7].

### Calculation of neighborhood variables

All spatial methods were performed with ArcGIS Pro 3.0 (ESRI, Redlands, CA) and other methods with RStudio [8]. Of the 150 survey participants in the dataset, 2 (1.3%) did not have a valid home addresses in NYC resulting in an analytic sample of 148 which were geocoded using ESRI’s world geocoding services. We obtained American Community Survey 2020 5-year estimates of median HHI (MHHI); race/ethnicity data by census block group (CBG) in NYC were acquired from the National Historic Geographic Information System [9]; these were then spatialized. Pedestrian-accessible routes were identified by filtering the Linear Integrated Ordered Network (LION) dataset from NYC Department of City Planning [10]. Pedestrian-accessible network buffers were then created by measuring ¼ mile (∼ 400m) along the network from each participant’s home location. Race/ethnicity for each participant’s “neighborhood” was then calculated using areal weighting [11], meaning that the area of the portion of each CBG which is intersected by the buffer is calculated, and the ratio of each intersected CBG area to total CBG area is used to weigh the population counts (e.g., if CBG “A” has 25% of its area within the buffer, we assume that 25% of its population is also within the buffer). The neighborhood MHHI was then calculated using population-weighted means based on the areal weighting results (e.g., if CBG “A” has 100 residents within the buffer and an MHHI of $10,000 and CBG “B” has 50 residents within the buffer and an MHHI of $40,000, the population-weighted mean MHHI would be ((100×$10,000) + (50×$40,000)) / (100 + 50) = $20,000. Areal and population weighting, in combination with the utilization of pedestrian-accessible network buffers, aid in reducing the impact of edge effect, the modifiable area unit problem, and other sources of geospatial-related error [12].

### Calculation of concordance/discordance for a categorical variable

To compare participant race/ethnicity with neighborhood-level characteristics, the majority race for each neighborhood was calculated (i.e., > 50% of one race/ethnicity). If there was no majority, it was coded as “No Majority.” Concordance was defined as when the individual’s race/ethnicity is the same as that of the majority group in the neighborhood and discordance when they were different from the majority group. Participants were coded as “Neutral” if there was no majority in the neighborhood.

### Calculation of concordance/discordance for a continuous variable

For comparing participant-reported HHI and neighborhood-level MHHI, we calculated quintiles for NYC based on CBG-level data. The break values for the quintiles (Q1, Q2, Q3, Q4, Q5) were then modified to match the nearest break values in the survey data, resulting in < $40,000 (Q1), $40,000 to < $60,000 (Q2), $60,000 to < 80,000 (Q3), $80,000 to < $100,000 (Q4), and ≥ $100,000 (Q5). These cutoffs were selected based on the data available from our survey and the distribution of HHI in NYC. Other cutoffs may be used for other variables, depending on the distribution (normal, skewed, biphasic, etc.) of that characteristic in the population being studied . Concordance was defined as individual and neighborhood HHI being in the same quintile (e.g., both participant and neighborhood HHI are in Q1) and discordance when there is more than one quintile between individual and neighborhood HHI (e.g., the individual’s HHI is in Q1, but the neighborhood HHI is in Q3, Q4, or Q5). Participants were coded as “Neutral” when their HHI quintile was only one class away from the neighborhood (e.g., participant in Q1 and neighborhood in Q2).

## Results

Table 1 displays the distribution of participant HHI and corresponding neighborhood MHHI. Notably, over 40% of participants reported HHIs of < $20,000, with over 60% reported < $40,000, which represents the upper limit of the first quintile in the analysis. It is worth mentioning that no participants reported HHIs in the fifth quintile (> $100,000).

**Table 1.** Individual- and neighborhood-level household income (n = 147*)

Participant household income	Quintile	*n* (%)	Neighborhood median household income
			(mean [SD])
<20k	Q1	60 (40.8)	48,960 (27, 225)
20–40k	Q1	31 (21.1)	48,532 (27, 260)
40–60k	Q2	17 (11.6)	48,560 (18, 929)
60–80k	Q3	7 (4.8)	70,816 (46, 146)
80–100k	Q4	15 (10.2)	95,127 (42, 787)
>100k	Q5	0 (0.0)	NA
Chose not to answer	NA	17 (11.6)	59,158 (23, 091)

* Three participants were excluded (2.0%). Two had invalid addresses, and one was in an area without reliable area-level median household income (MHHI) data.

Table 2 illustrates the relationship between participant and neighborhood race/ethnicity. The majority of participants were either Hispanic/Latino (48.0%) or non-Hispanic (NH) Black (39.2%).

**Table 2.** Individual- and neighborhood-level race/ethnicity (n = 148*)

Participant race/ethnicity		Neighborhood race/ethnicity (mean [SD])				
	*n* (%)	% Hispanic	% NH Black	% NH Asian	% NH other	% NH White
Hispanic/Latino	71 (48.0)	47.13 (16.96)	28.75 (13.93)	7.09 (7.47)	2.49 (1.61)	14.54 (16.57)
NH Black	58 (39.2)	40.72 (17.80)	35.85 (17.50)	5.71 (5.43)	2.95 (2.20)	14.76 (14.65)
NH Other	6 (4.1)	39.57 (20.83)	21.77 (19.48)	13.02 (11.80)	2.95 (2.18)	22.70 (19.48)
NH White	13 (8.8)	26.79 (22.53)	10.91 (11.69)	10.58 (8.04)	3.57 (2.58)	48.17 (26.45)

NH = non-Hispanic.

* Two participants were excluded (1.3%) due to invalid addresses.

Table 3 presents the relationship between participant-reported HHI and neighborhood-level MHHI by quintile. Overall, nearly 20% of participants live in neighborhoods discordant with their individual HHI.

**Table 3.** Concordance and discordance of household income (n = 130*)

Individual HHI quintile	Neighborhood MHHI quintile (N [row %])					Concordant (*N* [row %])	Neutral (*N* [row %])	Discordant (*N* [row %])
	Q1	Q2	Q3	Q4	Q5			
Q1	36 (39.56)	39 (42.86)	5 (5.49)	5 (5.49)	6 (6.59)	36 (39.56)	39 (42.86)	16 (17.58)
Q2	8 (47.06)	4 (23.53)	3 (17.65)	2 (11.76)	0 (0)	4 (23.53)	11 (64.71)	2 (11.76)
Q3	2 (28.57)	2 (28.57)	1 (14.29)	1 (14.29)	1 (14.29)	1 (14.29)	3 (42.86)	3 (42.86)
Q4	0 (0)	4 (26.67)	2 (13.33)	1 (6.67)	8 (53.33)	1 (6.67)	10 (66.67)	4 (26.67)
*Total*	*46 (35.38)*	*49 (37.69)*	*11 (8.46)*	*9 (6.92)*	*15 (11.54)*	*42 (32.31)*	*63 (48.46)*	*25 (19.23)*

* Twenty participants (13.3%) were excluded due to choosing not to answer the income question (*n* = 17), invalid addresses (*n* = 2), or unreliable neighborhood median household income (MHHI) data (*n* = 1).

Table 4 depicts the relationship between participant race/ethnicity and neighborhood racial/ethnic composition. Approximately 22% of participants lived in neighborhoods with a different racial/ethnic majority than their own, primarily driven by NH Black participants, 31% of whom reside in predominantly Latino/Hispanic neighborhoods.

**Table 4.** Concordance and discordance of race/ethnicity (n = 148*)

Individual race/ethnicity	Neighborhood majority race/ethnicity (*N* [row %])				Concordant (*N* [row %])	Neutral (*N* [row %])	Discordant (*N* [row %])
	Hispanic/Latino	NH Black	NH White	No Majority			
Hispanic/Latino	34 (47.89)	4 (5.63)	4 (5.63)	29 (40.85)	34 (47.89)	29 (40.85)	8 (11.27)
NH Black	18 (31.03)	14 (24.14)	2 (3.45)	24 (41.38)	14 (24.14)	24 (41.38)	20 (34.48)
NH Other	3 (50)	1 (16.67)	0 (0)	2 (33.33)	0 (0)	2 (33.33)	4 (66.67)
NH White	2 (15.38)	0 (0)	9 (69.23)	2 (15.38)	9 (69.23)	2 (15.38)	2 (15.38)
*Total*	*57 (38.51)*	*19 (12.84)*	*15 (10.14)*	*57 (38.51)*	*57 (38.51)*	*57 (38.51)*	*34 (22.97)*

NH = non-Hispanic.

* Two participants were excluded (1.3%) due to invalid addresses.

## Discussion

This manuscript introduces a methodological approach to operationalizing the concepts of concordance and discordance between individual characteristics and neighborhood-level social determinants. Beyond relying solely on the American Community Survey, an expanding array of publicly accessible neighborhood-level data sources exists. The outlined approach, applied here to compare self-reported household income with neighborhood-level MHHI, could be adapted for any bi-level SDoH measured continuously, such as annual income, net worth, education level, age, household size, or duration of residence at the same address. The specification of “concordant” and “discordant” may be varied, and a designation of “neutral” may or may not be included, based on either empirical or conceptual considerations. Similarly, the methodology for race/ethnicity could be extended to other categorical variables like preferred language, country of origin, employment status, homeownership, health insurance, and housing type; comparable neighborhood-level measures are available for all of these and more. (See Table 5.) In our example, we set the concordance threshold at > 50%, which we deemed appropriate for race/ethnicity; however, alternative thresholds could be chosen, with lower thresholds possibly more suitable for other social determinant domains.

**Table 5.** Comparable neighborhood-level social determinant of health measures

SDoH domains	Individual-level variables	Neighborhood-level variables
Demographic ccharacteristics	Race: Black, Native	% Black, not Hispanic
	American, Alaskan or	% Asian, not Hispanic
	Hawaiian, Asian	% Hispanic/Latino
	Ethnicity: Hispanic/	% White, not Hispanic
	Latino disability	% Disabled
Education	Less than high school	% Less than HS
Economic stability	Financial strain	% On family assistance
	Employment food	% Unemployed
	Insecurity	% Food
	Housing stability	Stamps/SNAP
		Food accessibility
		% Home ownership
	Income	Eviction rates
		Median household
		income
Sociocultural environment	Country of origin	% Foreign born
	Preferred language	% Limited English
		proficiency
	Household composition	Mean household size
		Housing density
	Incarceration	
		% Incarcerated
Physical/built environment	Physical activity	# Running tracks
		# Parks, walking paths
		Air quality
	Smoking	
	Transportation	Proximity to subway/bus
		Assault hospitalizations
	Exposure to violence	Felony: violent crimes
Sociocultural environment	Health insurance	% Uninsured
	Self-rated health	
	Primary care provider	Primary care physicians per capita

Since neighborhoods encompass multiple social determinants, individuals may reside in neighborhoods that exhibit concordance on some factors but discordance on others. Residential settlement patterns may be driven by social preferences related to race, ethnicity, national origin, immigration status, or age. Observable patterns of concordance and discordance may reflect these social preferences or may be driven by specific sociodemographic phenomena, such as early- and late-stage gentrification scenarios. Given the association between race/ethnicity and numerous social risks, certain combinations of concordance and discordance could give rise to social phenotypes that are linked to distinct health risks and outcomes. By operationalizing and quantifying concordance and discordance, the proposed method facilitates the exploration of these patterns and their health impacts.

As previously discussed, interactions between discordant individual-level factors and neighborhood-level factors may influence health outcomes at both individual and neighborhood levels. For instance, individuals with higher education levels might adopt protective behaviors that mitigate the health effects of adverse neighborhood characteristics, or they may foster neighborhood-level collective efficacy and social capital through community engagement, leading to broader health benefits [13,14]. Future research employing our definitions of concordance and discordance could concurrently investigate social determinants at both individual and neighborhood levels to uncover potential interactions.

### Limitations

The data presented here serve solely to illustrate the proposed operational definitions of concordance and discordance. Our current cohort contains 150 participants; a larger planned study will recruit a total of 2800 participants over 5 years. Our recruitment sites were in an urban setting; the definition of neighborhood we used for this densely populated area would have to be modified for a rural setting. As with any self-reported data, our survey responses are subject to numerous biases including social desirability, recall, response, cognitive, sampling, social context, response set, acquiescence, order effect, and language/cultural biases. These biases can distort data accuracy and reliability, impacting research validity. While racially and ethnically diverse, our sample is skewed toward individuals with lower household incomes, reflecting their disproportionate utilization of ED services which is consistent to current literature [15].

However, as the purpose of this study is to demonstrate our proposed approach to operationalize concordance and discordance between individual and neighborhood social determinant, we performed our analysis with the currently available data. Our future study will recruit a larger cohort with paired individual- and neighborhood-level data. This will allow us to broaden our analyses to include additional social determinants and to accurately delineate patterns of concordance and discordance between individual and neighborhood characteristics and their impacts on health outcomes. Finally, we encourage future studies to replicate our methodology in larger and more diverse cohorts to corroborate our findings and strengthen the evidence base.

## Conclusion

Individual- and neighborhood-level social determinants confer both risks and benefits that influence health outcomes. The proposed method offers an approach to defining and quantifying concordance and discordance between individual characteristics and neighborhood-level social determinants. This methodology will facilitate more robust investigations needed to fully grasp the intricate interplay between individuals and their environments and to accurately model the true impacts of social determinants on health.

## Supporting information

Ngai et al. supplementary material Ngai et al. supplementary material

## Supplementary material

The supplementary material for this article can be found at https://doi.org/10.1017/cts.2024.517.
