# Supplementary material for: Measuring concordance and discordance between selected individual characteristics and corresponding neighborhood-level social determinants of health
Source: J Clin Transl Sci. 2024 Apr 15;8(1):e77. doi: 10.1017/cts.2024.517 (PMC11075104; doi:10.1017/cts.2024.517)
Supplement: Ngai et al. supplementary material [file S205986612400517Xsup001.pdf]

Priority Survey Enrollment Survey

Please complete the survey below.

Thank you!

Are you Hispanic or Latino?
- ◯ Yes
- ◯ No
- ◯ I chose not to answer this question

(Ethnicity)

Which race(s) are you? Check one unless you identify as mixed race, then check all that apply.
- ☐ Non-Hispanic White
- ☐ Black/African-American
- ☐ Native Hawaiian
- ☐ American Indian/Alaska Native
- ☐ Asian
- ☐ Pacific Islander
- ☐ Other
- ☐ I chose not to answer this question

What is your gender?
- ☐ Female
- ☐ Male
- ☐ Trans male/Trans man
- ☐ Trans female/Trans woman
- ☐ Gender queer/gender non-confirming/non-binary
- ☐ I choose not to answer this question

What year were you born?

____

Were you born in the United States?
- ◯ Yes
- ◯ No

If not, where were you born?

____

What language are you most comfortable speaking?
- ☐ English
- ☐ Spanish
- ☐ Other Language
- ☐ I chose not to answer

What is the highest level of education that you have finished?
- ☐ less than a high school diploma
- ☐ High School diploma or GED
- ☐ Some college or trade school
- ☐ College degree
- ☐ Masters or doctoral degree
- ☐ I chose not to answer this question

	No, no difficulty	Yes, some difficulty	Yes a lot of difficulties	Cannot do at all
Difficulty seeing, even if wearing glasses	◯	◯	◯	◯

Difficulty hearing, even if using a hearing aid?	◯	◯	◯	◯
Difficulty walking or climbing steps?	◯	◯	◯	◯
Difficulty remembering or concentrating?	◯	◯	◯	◯
Difficulty taking care of yourself- doing things like washing all over or dressing?	◯	◯	◯	◯

FAMILY

How many family members, including yourself, do you currently live with?

◯ 1
◯ 2
◯ 3
◯ 4
◯ 5
◯ 6
◯ 7
◯ 8
◯ 9
◯ 10

Do you live with an older adult (aged 65 years of age or older)?

◯ Yes
◯ No

Do you live with a child/children?

◯ Yes
◯ No

HOUSING

What is your housing situation today?

◯ I/We live in a home that I/we own
◯ I/We live in a home that I/we rent
◯ I do not have housing (staying with others, in a hotel, in a shelter, living outside on the street, on a beach, in a car or in a park)
◯ I choose not to answer this question

Are you worried about losing your housing?

◯ No
◯ Yes
◯ I chose not to answer

What address do you live at? (Street) (e.g 123 Main Street)

____

Zip code:
(NYC residents only)

____

Do you feel physically and emotionally safe where you currently live?

◯ No
◯ Yes
◯ Unsure
◯ I choose not to answer this question

In the last 12 months, how many times have you moved from one home to another?

____

In the past year, have you spent more than 2 nights in a row in a jail, prison, detention center, or juvenile correctional facility?	◯ No<br>◯ Yes<br>◯ I chose not to answer this question

EMPLOYMENT

What is your current work situation?	◯<br>◯ Unemployed<br>◯ Part-time or temporary work<br>◯ Full time work<br>◯ Not employed but not seeking work (e.g student, retired, disabled, unpaid primary care giver)<br>I choose not to answer this question
If employed, do you work outside the home?	◯ Yes<br>◯ No
Do you work remotely?	◯ Yes<br>◯ No
What industry do you work in?	◯ Healthcare<br>◯ Retail<br>◯ Education<br>◯ First Responder<br>◯ Criminal Justice<br>◯ Public Transit<br>◯ Direct Service Provider<br>◯ Other
If Other, what industry? Name industry here:	__________________________________
What is your main health insurance?	◯ None/Uninsured<br>◯ Commercial insurance<br>◯ Medicaid<br>◯ Medicare<br>◯ Unsure/Don't know<br>◯ I chose not to answer this question
During the past year, what was the total combined household income for you and the family members you live with?	◯ Under or over $20,000<br>◯ Under or over $40,000<br>◯ Under or over $60,000<br>◯ Under or over $80,000<br>◯ Under or over $100,000<br>◯ I choose not to answer this question

In the past year, have you or any family members you live with being unable to get any of the following when it was really needed? Check all that apply

	No	Yes
Food	◯	◯
Clothing	◯	◯
Utilities	◯	◯
Child Care	◯	◯
Phone	◯	◯

Medicine or Any Health care (Medical, Dental, Vision)	◯	◯
I chose not to answer this question	◯	◯

How hard is it for you to pay for the very basics like food, housing, heating, medical care and, and medications?

◯ Not hard at all
◯ Somewhat hard
◯ Very hard
◯ I chose not to answer this question

Has lack of transportation kept you from medical appointments, meetings, work or from getting things needed for daily living?

◯ No
◯ Yes, it has kept me from medical appointments or from getting my medications
◯ Yes, it has kept me from non-medical meetings, appointments, work, or getting things needed for daily living
◯ I choose not to answer this question

Physical, Social and Emotional Health

On average, how many days per week do you engage in moderate to strenuous exercise (like walking fast, running, jogging, dancing, swimming, biking, or other activities that cause light or heavy sweat?)

◯ 0
◯ 1
◯ 2
◯ 3
◯ 4
◯ 5
◯ 6
◯ 7

Do you smoke cigarettes or e-cigarettes every day, some days, or not at all?

◯ Every day
◯ Some days
◯ Not at all
◯ Don't know/Not Sure
◯ Refused

In general, would you say your health is:

◯ Excellent
◯ Very good
◯ Good
◯ Fair
◯ Poor

Do you have a primary care provider?

◯ Yes
◯ No

During the past 12 months, how many times have you seen a doctor or other health care professional about your health at a doctor's office, a clinic, or some other place?

◯ 0
◯ 1
◯ 2 to 3
◯ 4 to 5
◯ 6 to 7
◯ 8 to 9
◯ 10-12
◯ 13-15
◯ 16 or more
◯ Refused
◯ Don't know

Do you have Asthma or COPD?	◯ Yes<br>◯ No
Do you have Hypertension?	◯ Yes<br>◯ No
Do you have Kidney Disease?	◯ Yes<br>◯ No
Do you have Diabetes?	◯ Yes<br>◯ No
Do you have Heart Failure?	◯ Yes<br>◯ No

PHYSICAL ABUSE

CRC Note: If patient is being abused, pleas offer social work consult after survey is complete

In the last 12 months, have you been physically or emotionally hurt or threatened by a spouse/partner or someone else you know?	◯ No<br>◯ Yes<br>◯ I chose not to answer this question
How often do you see or talk to people that you care about and feel close to?	◯ less than once a week<br>◯ 1 or 2 times a week<br>◯ 3 or 5 times a week<br>◯ 5 or more times a week<br>◯ I chose not to answer this question

STRESS

If patient says he/she is very stressed, offer social work consult after survey is complete.

Stress is when someone feels tense, nervous, anxious, or can't sleep at night because their mind is troubled. How stressed are you?	◯ Not at all<br>◯ A little bit<br>◯ Somewhat<br>◯ Quite a bit<br>◯ Very much<br>◯ I chose not to answer this question

Loneliness

CRC note: If the patient feels isolated or lonely, please offer a social work consult after the survey is complete.

How often do you feel lonely or isolated from those around you?

- ◯ Never
- ◯ Rarely
- ◯ Somewhat
- ◯ Often
- ◯ Always

How often have they been bothered by the following over the past 2 weeks?

	Not at all	Several days	More than half of the days	Nearly every day
Little interest or pleasure in doing things?	◯	◯	◯	◯
Feeling down, depressed, or hopeless?	◯	◯	◯	◯

CRC NOTE: IF patient says yes to experiencing discrimination, offer a social work consult

Have you ever experienced discrimination, or been prevented from doing something, or been hassled or made to feel inferior in any of the following situations because of your race, ethnicity, or color?

	No	Yes
At school?	◯	◯
Getting hired or getting a job?	◯	◯
At work?	◯	◯
Getting housing?	◯	◯
Getting medical care?	◯	◯
Getting service in a store or restaurant?	◯	◯
Getting credit, bank loans, or a mortgage?	◯	◯
On the street or in a public setting?	◯	◯
From the police or in the courts?	◯	◯

Would you like to talk with a social worker?

- ◯ Yes
- ◯ No
